# Long‐Term Real‐World Outcomes of Primary CNS Lymphoma Patients Treated With MATRix Regimen Are Similar to IELSG32 Trial Results

**DOI:** 10.1002/hon.70142

**Published:** 2025-10-18

**Authors:** Prokop Vodicka, Andrea Janikova, David Belada, Veronika Hanackova, Heidi Mocikova, Juraj Duras, Katerina Steinerova, Katerina Benesova, Eva Konirova, Tomas Prochazka, Kamila Polgarova, Michal Masar, Jitka Dlouha, Petra Blahovcova, Marek Trneny

**Affiliations:** ^1^ First Department of Medicine First Faculty of Medicine Charles University General Hospital Prague Czech Republic; ^2^ Department of Hematology and Oncology Faculty of Medicine Masaryk University and University Hospital Brno Czech Republic; ^3^ Fourth Department of Internal Medicine—Hematology Faculty of Medicine Charles University and University Hospital Hradec Kralove Hradec Králové Czech Republic; ^4^ Department of Haemato‐Oncology Faculty of Medicine and Dentistry Palacky University and University Hospital Olomouc Czech Republic; ^5^ Department of Hematology Third Faculty of Medicine Charles University and University Hospital Kralovske Vinohrady Prague Czech Republic; ^6^ Department of Hematology Medical Faculty of the Ostrava University and University Hospital Ostrava Czech Republic; ^7^ Department of Haematology and Oncology Faculty of Medicine Charles University and University Hospital Pilsen Pilsen Czech Republic; ^8^ Datacenter Czech Lymphoma Study Group Prague Czech Republic

**Keywords:** autologous stem cell transplantation, IELSG32 trial, MATRix regimen, primary central nervous system lymphoma, real‐world results

## Abstract

Primary central nervous system lymphomas (PCNSL) are rare malignancies with poor survival outcomes. The IELSG32 trial demonstrated efficacy of MATRix chemoimmunotherapy followed by autologous stem cell transplantation (auto‐SCT) in PCNSL patients aged ≤ 70 years with a performance status (PS) ECOG ≤ 3. However, long‐term real‐world results of MATRix/auto‐SCT therapy remain limited. This analysis, with a median follow‐up of 52 months, aimed to evaluate the outcomes of MATRix‐treated PCNSL patients in clinical practice. From 2015 to 2022, 280 PCNSL patients who received systemic therapy were identified in the NiHiL project (NCT03199066). Eighty‐eight individuals treated with MATRix entered the analysis. Endpoints included efficacy and safety of induction and consolidation therapy. Seventy‐eight patients who met key IELSG32 inclusion criteria (age ≤ 65 years and PS ECOG ≤ 3, or age 66–70 years and PS ECOG ≤ 2) achieved an overall response rate of 82% (complete remission rate 58%) following MATRix regimen. After median follow‐up of 52 months, 4‐year progression‐free survival and overall survival (OS) rates were 53% and 55%, respectively. Forty‐six (59%) patients completed MATRix treatment, and 32 (41%) discontinued induction therapy (15 toxicity, 11 infections, 5 progressive diseases, 1 refusal). The treatment‐related mortality was 8%. Among 67 patients with responsive/stable disease, 50 underwent consolidation with whole‐brain radiotherapy (WBRT, *n* = 13) or auto‐SCT (*n* = 37). No significant survival differences were observed between WBRT and auto‐SCT (4‐year OS 84% vs. 74%, HR 0.61, 95% CI 0.16–2.29, *p* = 0.467). Long‐term real‐world outcomes of MATRix/auto‐SCT therapy are comparable to IELSG32, supporting its use in younger, fit PCNSL patients.

**Clinical Trial Registration:**

The data in this analysis were collected in the observational Czech non‐Hodgkin lymphoma registry “NiHiL” (NCT03199066)

## 1 Introduction

Primary central nervous system lymphomas (PCNSL) are rare hematologic malignancies, representing up to 4% of non‐Hodgkin lymphomas and 6% of extranodal lymphomas [1, 2]. Histologically, diffuse large B‐cell lymphomas (DLBCL) represent the majority of PCNSL (> 90%), while other lymphoma subtypes occur rarely [1]. Along with vitreoretinal and testicular lymphomas, PCNSL is now classified under the new umbrella of “primary large B‐cell lymphoma of immune‐privileged sites,” characterized by shared molecular features such as activated B‐cell origin and frequent *MYD88* mutations [3, 4].

Despite advances in therapies for systemic non‐Hodgkin lymphoma, innovative treatments for PCNSL remain limited, and the prognosis of PCNSL has been considered to be poor [5, 6]. Standard treatment of PCNSL includes induction therapy with chemotherapeutic agents capable of crossing the blood‐brain barrier, typically high‐dose methotrexate‐based regimens. Patients who respond to induction treatment proceed to consolidation therapy, including either whole‐brain radiotherapy (WBRT), or high‐dose chemotherapy followed by autologous stem cell transplantation (auto‐SCT) for patients fit enough to undergo this procedure [2, 7].

The randomized phase II International Extranodal Lymphoma Study Group 32 (IELSG32) trial compared three different chemotherapeutic regimens for PCNSL patients aged ≤ 70 years demonstrating that the combination of methotrexate, cytarabine, thiotepa, and rituximab (i.e., MATRix regimen) is the most effective induction therapy. The use of MATRix regimen resulted in significantly improved long‐term outcomes, with a 7‐year overall survival (OS) of 56% [8]. Eligible patients in the trial were further randomized to consolidation with either WBRT or auto‐SCT. Both approaches showed similar efficacy; however, patients undergoing auto‐SCT experienced improved neurocognitive outcomes [9, 10]. As a result, the MATRix induction regimen with auto‐SCT consolidation is now the recommended standard of care for fit PCNSL patients [11]. Despite its clinical adoption, data on the long‐term efficacy and safety of the MATRix regimen in real‐world settings remain limited [12, 13].

This study aimed to evaluate the real‐world outcomes of MATRix‐treated PCNSL patients who met the IELSG32 inclusion criteria (IC) compared to the IELSG32 trial results. Additionally, we aimed to investigate the efficacy of MATRix therapy in clinical practice among patients meeting versus not meeting the IELSG32 IC.

## 2 Methods

### 2.1 Patients' Selection

Consecutive patients with newly diagnosed PCNSL, treated and followed up at seven university centers in the Czech Republic were prospectively collected in the non‐Hodgkin lymphoma “NiHiL” project (NCT03199066). All patients signed a written informed consent, and the study was approved by the local ethics committees.

**FIGURE 1:**
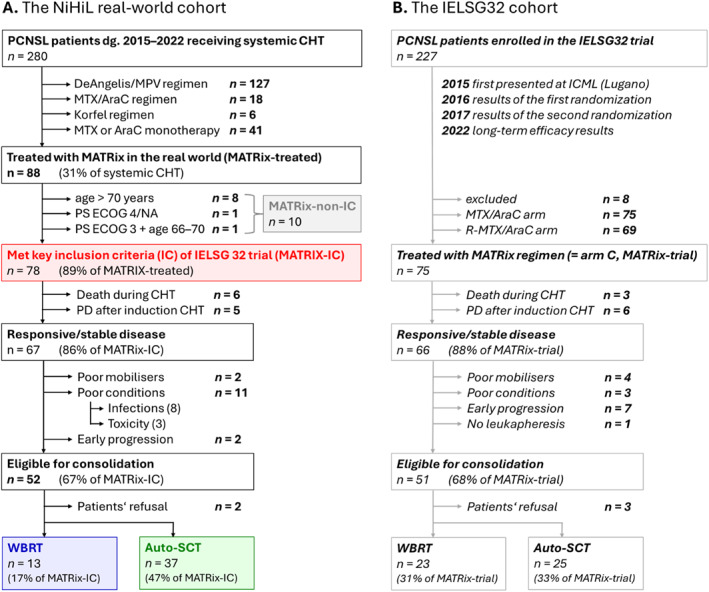
Patients flow chart. Auto‐SCT, autologous stem cell transplantation; CHT, chemotherapy; IC, inclusion criteria; PCNSL, primary central nervous system lymphoma; WBRT, whole brain radiotherapy.

During the screening period between January 2015 (introduction of MATRix regimen into routine clinical practice in the Czech Republic) and December 2022, 290 PCNSL patients with histologically or cytologically confirmed DLBCL of CNS with parenchymal involvement and with no evidence of current or prior systemic lymphoma involvement (confirmed by positron emission tomography/computed tomography scans and bone marrow biopsy) were identified in the NiHiL registry. Of these, 280 individuals received systemic induction chemotherapy (Figure 1, Supporting Information S1). Treatment selection followed current PCNSL treatment guidelines and local clinical practices.

### 2.2 Induction Therapy

Among 280 patients receiving systemic induction therapy, 88 (31%) patients who were intended to and received at least one cycle of the MATRix regimen entered the analysis, all outside the IELSG32 study. All 88 included patients were tested for human immunodeficiency virus and tested negative. During therapy, patients received granulocyte colony stimulating factors and anti‐infectious prophylaxis according to local guidelines.

Baseline characteristics of the 88 MATRix‐treated PCNSL patients were prospectively collected in the NiHiL registry and verified from the medical records of each participant. This includes age, gender, performance status according to the Eastern Cooperative Oncology Group (PS ECOG) [14], and Memorial Sloan Kettering Cancer Center (MSKCC) index [15].

Of these, 78 (89%) MATRix‐treated individuals fulfilled the two key IC of the IELSG32 trial.

1. Age 18–65 years with PS ECOG 0–3, or
2. Age 66–70 years with PS ECOG 0–2

These patients were included in the analysis as MATRix‐IC cohort.

The remaining 10 (11%) patients didn't meet the IC due to age over 70 years (*n* = 8), PS ECOG 4 (*n* = 1), or PS ECOG 3 in individuals aged 66–70 years (*n* = 1), and entered the analysis as MATRix‐non‐IC cohort.

### 2.3 Consolidation Therapy

Patients with a responsive or stable disease after induction MATRix chemoimmunotherapy were evaluated for consolidation therapy eligibility. Consolidation options included WBRT, or high‐dose chemotherapy followed by auto‐SCT, determined by institutional practices, patient fitness and preferences. Randomization between the two approaches was not performed.

### 2.4 Response Rates

Therapy responses were assessed using gadolinium‐enhanced magnetic resonance imaging (MRI) of the brain after induction therapy, post‐consolidation therapy (if applicable), and during follow‐up. Responses were categorized as overall response rate (ORR), complete remission (CR), partial remission (PR), stable disease (SD), progressive disease (PD), or not assessed (NA) [16].

### 2.5 Objectives and Endpoints

The objective of this analysis was to describe the outcomes of PCNSL patients meeting the IELSG32 trial IC and receiving MATRix regimen in the real world, and to compare these results with the IELSG32 trial. In detail, the endpoints of this analysis were the following.

1. Comparing response rates and progression‐free survival (PFS) between the MATRix‐IC cohort in this study and MATRix‐treated patients in the IELSG32 trial (arm C) after induction therapy, regardless of consolidation (i.e., endpoints after the first randomization of the respective trial),
2. Comparing overall survival (OS), PFS and response rates of MATRix‐IC patients receiving consolidation with similarly treated IELSG32 trial patients (i.e., endpoints after the second randomization of the respective trial),
3. Comparing response rates, PFS, and OS between the MATRix‐non‐IC and MATRix ‐IC patients,
4. Comparing outcomes with two previously published real‐world datasets [12, 13].

### 2.6 Statistical Analysis

Categorical variables were described as counts and percentages, continuous variables were summarized using medians and ranges. Differences between categorical variables were assessed by Pearson's χ^2^ or Fisher's Exact Test. The Mann‐Whitney *U* test was applied for comparison between continuous variables. Survival analyses were conducted using the Kaplan‐Meier method and the Cox proportional hazard regression, with results presented as hazard ratios (HR) and 95% confidence intervals (CI). The PFS was defined as the time from diagnosis to relapse, progression, death, or last follow‐up. The OS was defined as the time from diagnosis to death, or last follow‐up. Statistical significance was set at *p* value < 0.05. All analyses were performed using *R* Statistical Software (v4.3.3; *R* Core Team 2024) and GraphPad Prism (version 8 for Windows, GraphPad Software, Boston, Massachusetts, USA).

## 3 Results

### 3.1 Baseline Characteristics

In the MATRix‐IC cohort (*n* = 78), the median age was 57 years (range 18–70 years), and 44 (56%) patients were male. PS ECOG 0–2 was observed in 67 (86%) of cases, and the low/intermediate‐risk MSKCC score in 70 (90%) patients. In the entire cohort of 88 MATRix‐treated PCNSL patients, the median age was 59.5 years (range 18–79 years), with 80 (91%) individuals aged up to 70 years and 51 (58%) being male. PS ECOG 0–2 was observed in 73 (83%) of cases, and a low/intermediate‐risk MSKCC score was recorded in 77 (87%) patients (Table 1).

**TABLE 1:** Baseline characteristics of patients with primary central nervous system lymphoma included in the analysis.

	MATRix‐ IC		MATRix‐ Non‐IC		*p* value	MATRix‐ Treated	
	*n*	%	*n*	%		*n*	%
No. of patients	78		10			88	
Age							
Median (range)	57	(18–70)	72.5	(40–79)	< 0.001^a^	59.5	(18–79)
< 50 years	23	29%	1	10%		24	27%
50–70 years	55	71%	1	10%		56	64%
> 70 years	0		8	80%		8	9%
Gender							
Male	44	56%	7	70%	0.412	51	58%
PS ECOG							
0	16	21%	1	10%	0.212	17	19%
1	26	33%	2	20%		28	32%
2	25	32%	3	30%		28	32%
3	11	14%	3	30%		14	16%
4	0		1	10%		1	1%
MSKCC score							
Low	23	30%	1	10%	0.135	24	27%
Intermediate	47	60%	6	60%		53	60%
High	8	10%	3	30%		11	13%
Intraocular^b^							
Yes	2	3%	0	0%	0.999	2	2%
LDH elevated							
Yes	38	49%	4	40%	0.603	42	48%

Abbreviations: IC, met IESLG32 inclusion criteria; MSKCC, Memorial Sloan Kettering Cancer Center; Non‐IC, did not met IESLG32 inclusion criteria; PS ECOG, performance status according to the Eastern Cooperative Oncology Group.

^a^ Marked statistically significant.

^b^ Concomitant to parenchymal involvement.

### 3.2 Induction Therapy

Among all chemotherapeutical regimens, the proportion of patients receiving MATRix chemotherapy increased over the inclusion period, from 19% in 2015 to 35% in 2022 (Supporting Information S1). In the MATRix‐IC cohort, a total of 64 individuals (82%) received corticosteroids prior to the MATRix initiation. The median number of MATRix cycles was 4 cycles (range 1–5), 46 (59%) patients completed the intended four cycles of therapy, while 32 (41%) discontinued the induction MATRix treatment. Across the entire MATRix‐treated cohort, the median number of induction cycles was four (range 1–5), with 47 (53%) patients receiving ≥ 4 cycles of the MATRix regimen.

The MATRix‐IC cohort demonstrated an ORR of 82% (*n* = 64) with a CR rate of 58% (*n* = 45), SD of 4% (*n* = 3), and PD of 8% (*n* = 6). The ORR after induction therapy for the entire MATRix‐treated cohort was 80% (*n* = 70), including a 58% CR rate (*n* = 51), 3% SD (*n* = 3), and 7% PD (*n* = 6).

**FIGURE 2:**
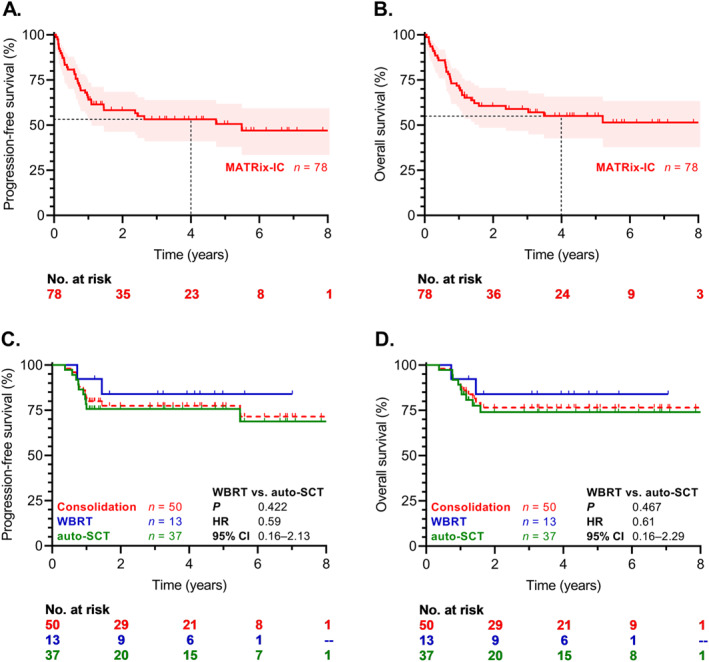
Survival curves of MATRix‐IC patients following induction (*n* = 78; (A) PFS, (B) OS) and consolidation therapy (*n* = 50; (C) PFS, (D) OS). Auto‐SCT, autologous stem cell transplantation; CI, confidence interval; HR, hazard ratio; IC, inclusion criteria; OS, overall survival; PFS, progression‐free survival; WBRT, whole brain radiotherapy.

The median follow‐up was 52 months (range 1.2–103 months). In the MATRix‐IC cohort (*n* = 78), the 2‐year PFS was 58%, and 4‐year PFS 53% (Figure 2A); the 2‐year OS was 61%, and 4‐year OS 55% (Figure 2B). Median PFS for the entire cohort of MATRix‐treated patients (*n* = 88) was 4.7 years, and the OS was 5.2 years (Supporting Information S1).

The most common reasons for MATRix treatment discontinuation among the 32 MATRix‐IC patients included toxicities (*n* = 15; 19%; hematologic *n* = 9, hepatotoxicity *n* = 3, renal *n* = 1, other/unknown *n* = 2), infectious complications (*n* = 11; 14%, most frequently febrile neutropenia in 7 cases), PD (*n* = 5; 6%), and patient refusal (*n* = 1; 1%). Out of the 26 patients who discontinued MATRix due to toxicity or infections, 7 (27%) patients were treated with different/less intensive treatment regimen (methotrexate, MPV or MTX/AraC regimen; with or without rituximab). In 5 of these 7 cases, this was followed by consolidation with WBRT among patients with responsive or stable disease. In addition, 4 patients (13%) underwent WBRT directly, without receiving any additional chemotherapy after MATRix discontinuation.

Overall, 6 (8%) MATRix‐IC patients died during induction therapy due to febrile neutropenia (*n* = 5) or other treatment‐related toxicity (*n* = 1), all occurring after the first cycle of MATRix regimen.

### 3.3 Consolidation Therapy

Altogether 67 (86%) patients in the MATRix‐IC cohort achieved either responsive or stable disease. Among them, eight individuals did not proceed to consolidation due to infectious complications during induction, 3 due to toxicity of induction therapy, 2 patients who were intended to auto‐SCT did not undergo the procedure due to insufficient mobilization of peripheral blood hematopoietic stem cells, and 2 patients experienced early PD before consolidation therapy. Ultimately, 52 (67%) patients were eligible for consolidation therapy; however, two individuals refused further treatment (Figure 1).

The consolidation treatment was administered to 50 patients (64%). The consolidation plan (auto‐SCT vs. WBRT) in the MATRix‐IC population was determined by the treatment center's algorithm. In total, 44 (56%) patients were initially planned for auto‐SCT, but 7 (9%) switched to WBRT due to toxicity during induction therapy. Finally, 37 (47%) patients with responsive or stable disease underwent auto‐SCT, and 13 (17%) received consolidative WBRT with a median total dose of 30 Gy (range 23–46 Gy).

The ORR after WBRT was 92% (*n* = 12 of 13), including 85% CR (*n* = 11 of 13); and after auto‐SCT, the ORR was 89% (*n* = 33 of 37), with 84% CR (*n* = 31 of 37; *p* = 0.681). There were no statistically significant differences in PFS or OS between WBRT and auto‐SCT recipients (4‐year PFS 84% vs. 74%, HR 0.59, 95% CI 0.16–2.13, *p* = 0.422, Figure 2C; 4‐year OS 84% vs. 74%, HR 0.61, 95% CI 0.16–2.29, *p* = 0.467, Figure 2D). Among the CR/PR/SD patients, the survival of consolidated individuals was superior to non‐consolidated patients (4‐year OS 77% vs. 24%, HR 0.19, 95% CI 0.06–0.60, *p* < 0.001); similar results were observed in CR only patients (4‐year OS 76% vs. 27%, HR 0.18, 95% CI 0.05–0.70, *p* < 0.001; Supporting Information S1).

### 3.4 Characteristics and Outcomes of the MATRix‐Non‐IC Cohort

In the MATRix‐non‐IC cohort (*n* = 10), the median age was 72.5 years (range 40–79 years), 7 (70%) patients were male. PS ECOG 0–2 was observed in 6 (60%) cases, and the low/intermediate‐risk MSKCC score in 7 (70%) patients. Patients in the MATRix‐non‐IC cohort were significantly older than those in the MATRix‐IC cohort (*p* < 0.001; Table 1). The median number of MATRix cycles was 2.5 cycles (range 1–4) with only three (30%) patients completing four cycles. The ORR was 60% (*n* = 6) with a CR rate of 60% (*n* = 6), and PD of 10% (*n* = 1). These patients tended to have shorter PFS compared to the MATRix‐IC cohort with 2‐year PFS 30% versus 58% (HR 2.93, 95% CI 0.99–8.75, *p* = 0.053); and significantly inferior OS with 2‐year OS 30% versus 61% (HR 3.14, 95% CI 1.04–9.52, *p* = 0.043; Supporting Information S1).

## 4 Discussion

The IELSG32 trial established MATRix induction followed by high‐dose chemotherapy and auto‐SCT as the preferred standard of care for fit PCNSL patients, with improved long‐term outcomes and better neurocognitive preservation compared with WBRT [8, 9, 10]. Our analysis extends these findings by providing long‐term data from the second‐largest real‐world cohort of MATRix‐treated patients, including those who did and did not meet IELSG32 inclusion criteria.

Key outcomes during induction treatment in our analysis were comparable to those reported in the IELSG32 trial, including response rates between real‐world MATRix‐treated PCNSL patients meeting key IELSG32 IC (i.e., age and PS ECOG) and the IELSG32 trial participants (ORR 82% vs. 88%; CR 58% vs. 49%, resp.) [8]. The incidence of treatment‐related mortality during induction was 8% (*n* = 6) in our analysis versus 4% in the trial population (*n* = 3). The PD rates at end‐of‐treatment restaging were similar (6% vs. 8%, resp.). Survival outcomes aligned closely, with a 4‐year PFS of 53% in our MATRix‐IC cohort, followed by a plateau in survival curves beyond this timepoint, compared to the 7‐year PFS of 52% observed in the IELSG32 MATRix arm [10]. Similarly, the 4‐year OS of 55% in our cohort was comparable to the 7‐year OS of 56% reported in that trial.

Regarding consolidation, 67% of patients in our MATRix‐IC cohort and 68% of IELSG32 MATRix‐treated patients were considered as being eligible for consolidation. In both cohorts, a small number of patients (two in our study and three in IELSG32) refused further therapy. The remaining patients (*n* = 50 vs. 48) underwent either auto‐SCT or WBRT. Auto‐SCT was preferentially used in our real‐world cohort (47% vs. 33% in the IELSG32 trial); conversely, the proportion of WBRT‐treated patients was lower (17% vs. 31%, resp.), reflecting the non‐randomized design of this real‐world analysis. Although survival outcomes were numerically higher for patients receiving WBRT compared to auto‐SCT (4‐year OS 84% vs. 74%), the difference was not statistically significant. These results are consistent with IELSG32 results (4‐year OS 85% vs. 83%), confirming that both consolidation strategies achieve similar efficacy following the MATRix regimen in real‐world and trial settings.

Patients not meeting the IELSG32 IC due to older age or reduced fitness experienced shorter survival compared to those who did meet the IC. Optimized treatment for this group remains undetermined, highlighting the need for reduced‐intensity regimens according to patients' overall fitness and comorbidities. Notably, a recent phase II MARTA trial (PMID 38301670) evaluating an age‐adapted intensified treatment approach in elderly and/or comorbid PCNSL patients has provided promising results, supporting the feasibility of tailored regimens in this challenging population [17].

Two real‐world studies have evaluated outcomes of MATRix‐treated PCNSL patients; however, none have provided long‐term follow‐up (Supporting Information S1) [12, 13]. One retrospective international study included 110 individuals who met IELSG32 IC with a median follow up of 27.4 months (vs. 52 months in our analysis) [12]. The analysis reported severe toxicities primarily during the first MATRix cycle, with a 5% mortality rate compared to 8% in our cohort. Treatment interruption was observed in 37% of their cases (vs. 41% in our study), with infectious complications accounting for 26% (compared to 28%). The ORR was 79% (vs. 80% in our cohort), and 68% (vs. 64%) proceeded to consolidation, with 48% (vs. 47%) undergoing auto‐SCT and 16% (vs. 17%) WBRT. The 2‐year PFS was 56% (vs. 58%), and OS was 64% (vs. 61%). Along with our data, patients not fulfilling the main IC of the IELSG32 trial (*n* = 46) had a significantly inferior outcome.

Another real‐world analysis of 37 MATRix‐treated patients with a median follow‐up time of 16.9 months reported an ORR of 83% after induction and a 2‐year PFS of 74% (78% in auto‐SCT recipients vs. 74%) [13]. These findings, consistent with our data, suggest the efficacy and tolerability of MATRix regimen are comparable across diverse real‐world populations.

Our study has several limitations. Center‐specific protocols may have introduced selection bias, favoring younger, fitter patients for MATRix and auto‐SCT. Although the efficacy and survival data were collected prospectively, the detailed data on toxicity and treatment discontinuation were extracted from medical records, which might underestimate their real frequency compared to IELSG32 trial. Additionally, post‐consolidation neurocognitive assessments were not performed.

Despite these limitations, our study provides several important observations. It includes a longer follow‐up period than other published real‐world analyses. Furthermore, our MATRix/auto‐SCT cohort is larger than the IELSG32 cohort. We also provide insights into the outcomes of a small group of patients who did not meet IELSG32 IC. Although the consolidation treatment allocation was non‐randomized, it reflects institutional practice, where persistent toxicity, poor mobilization, or patient refusal often compel treating physicians to choose WBRT. Importantly, along with the IELSG32 trial results, our findings suggest that WBRT can achieve outcomes similar to auto‐SCT, supporting its role as a viable therapeutic option.

In conclusion, with the longest median follow‐up time among real‐world studies, this analysis confirms the efficacy of the MATRix induction regimen followed by the auto‐SCT consolidation for young and fit PCNSL patients in real‐world settings, with outcomes comparable to IELSG32 and other real‐world cohorts. However, the high incidence of treatment discontinuation due to toxicities and infectious complications in nearly half of MATRix‐treated patients emphasizes the role of supportive care and careful patient selection for this intensive induction regimen. Outcomes for older and less fit patients remain poor, underscoring the need for alternative, less intensive treatment strategies tailored to their clinical status.

## Ethics Statement

Ethics approval was obtained from the local ethics committee and approved by the competent national authority. The study was conducted in accordance with the rules of Good Clinical Practice and the principles of the Declaration of Helsinki.

## Consent

All patients signed a written informed consent prior to the enrollment.

## Conflicts of Interest

The authors declare no conflicts of interest.

## Peer Review

The peer review history for this article is available at https://www.webofscience.com/api/gateway/wos/peer-review/10.1002/hon.70142.

## Supporting information


Supporting Information S1

## Data Availability

The datasets analyzed in this study are available from the corresponding author upon reasonable request. The corresponding author had full access to all data in the study and takes responsibility for the integrity of the data and the accuracy of the data analysis.
