# Supplementary material for: Long‐Term Real‐World Outcomes of Primary CNS Lymphoma Patients Treated With MATRix Regimen Are Similar to IELSG32 Trial Results
Source: Hematol Oncol. 2025 Oct 18;43(6):e70142. doi: 10.1002/hon.70142 (PMC12535275; doi:10.1002/hon.70142)

**SUPPLEMENTARY MATERIAL**

**Title:** Long-Term Real-World Outcomes of Primary CNS Lymphoma Patients Treated with MATRix Regimen are Similar to IELSG32 Trial Results

**Supplementary Table 1.** Summary of key outcomes in the NiHiL MATRix-IC patient cohort, IELSG32 trial MATRix-treated cohort (arm C) and two published real-world studies.

|  |  | **NiHiL**  **MATRix-IC** | **Schorb et al**  **(2020)^12^** | **Suleman et al**  **(2023)^13^** | ***IELSG32 trial (arm C)*^8-10^** |
| --- | --- | --- | --- | --- | --- |
| **No. of patients** | | 78 | 110 | 37 | *75* |
| **Auto-SCT consolidation** | | 37 (47%) | 53 (48%) | 22 (59%) | *25 (33%)* |
| **Follow-up (median months)** | | **52** | **27.4** | **16.9** | *88* |
| **Progression-free survival** | at 2 years | 58% | 56% | 54% | *61%* |
|  | at 4 years | 53% | --- | --- | *---* |
|  | at 7 years | --- | --- | --- | *52%* |

*Auto-SCT, autologous stem cell transplantation; IC, inclusion criteria.*

**Supplementary Figure 1.** Chemotherapy regimens used in the treatment of patients with primary central nervous system lymphoma (*n* = 280) during the inclusion period between 2015 and 2022 according to the NiHiL project data.


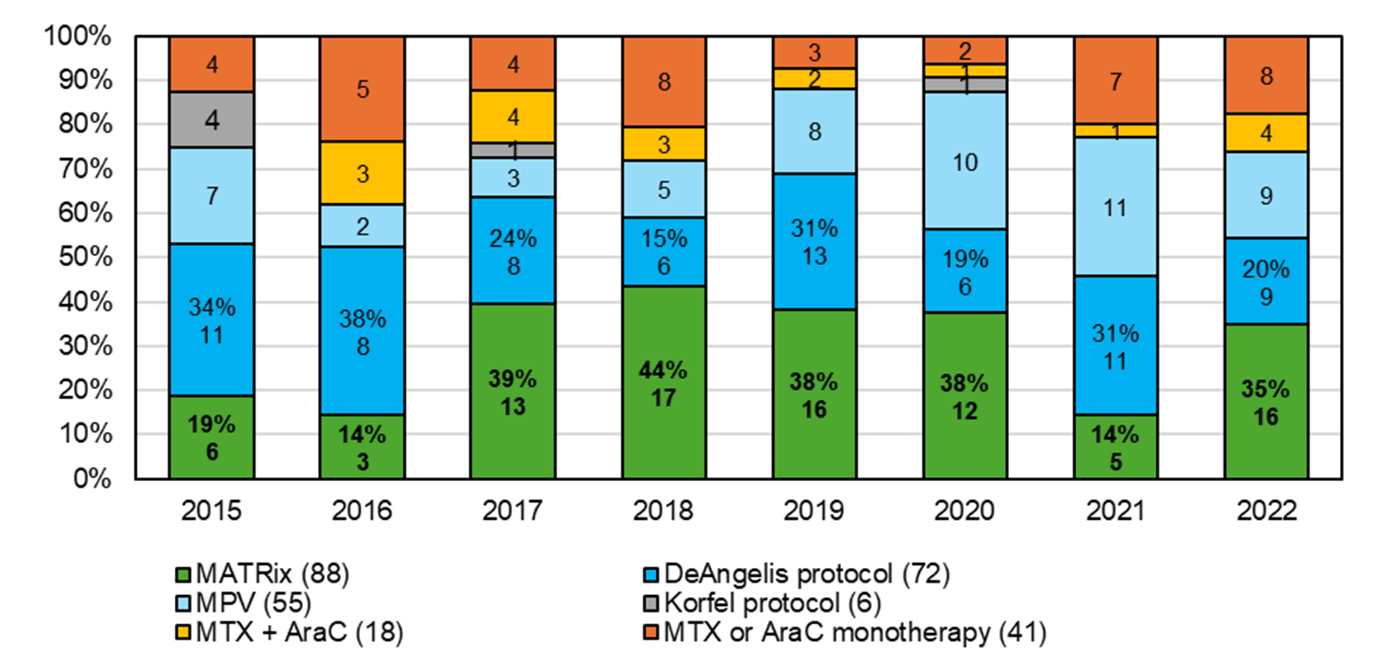


**Supplementary Figure 2.** Survival of the MATRix-treated patients (*n* = 88) included in the analysis (**A.** progression-free survival; **B.** overall survival).


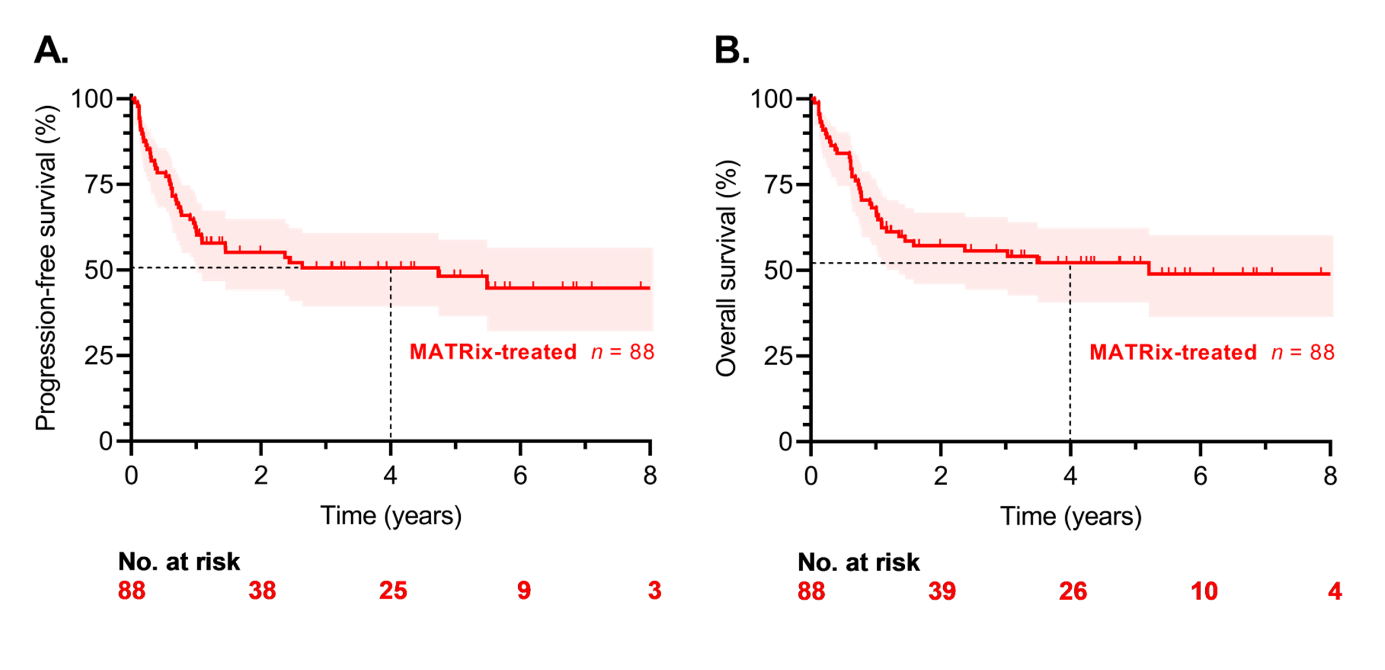


**Supplementary Figure 3.** Survival of the MATRix-IC patients receiving consolidation therapy (*n* = 50) versus those from the MATRix-IC cohort who were not consolidated (*n* = 15) among individuals with responsive or stable disease after exclusion of patients with early progressive disease (**A**, **B**), and of patients who reached complete remission (*n* = 36 versus 9) after induction therapy (**D**, **E**).

*CI, confidence interval; HR, hazard ratio; IC, inclusion criteria.*


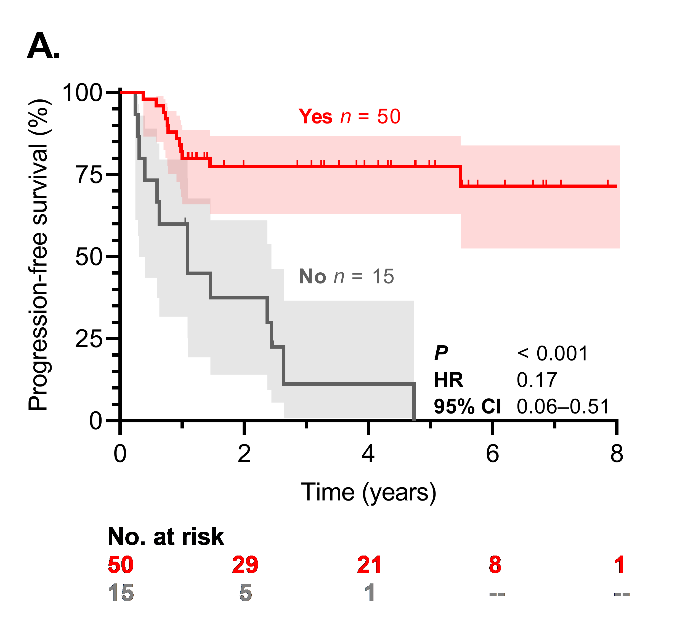

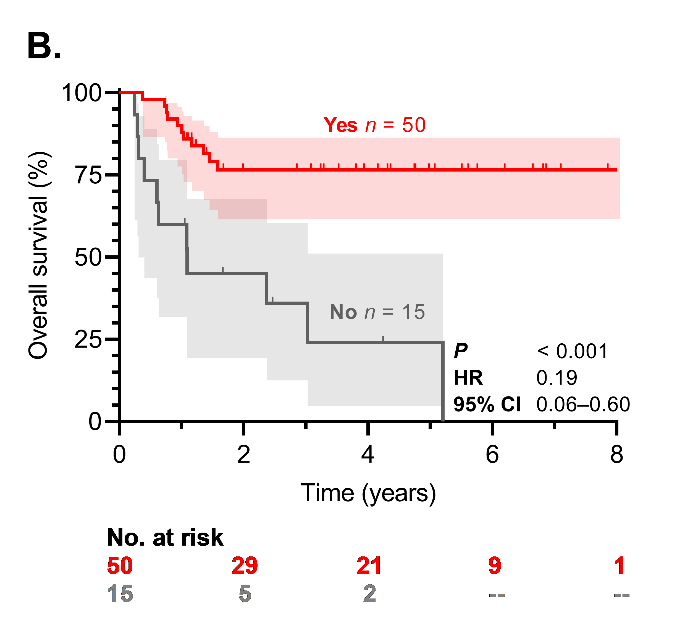


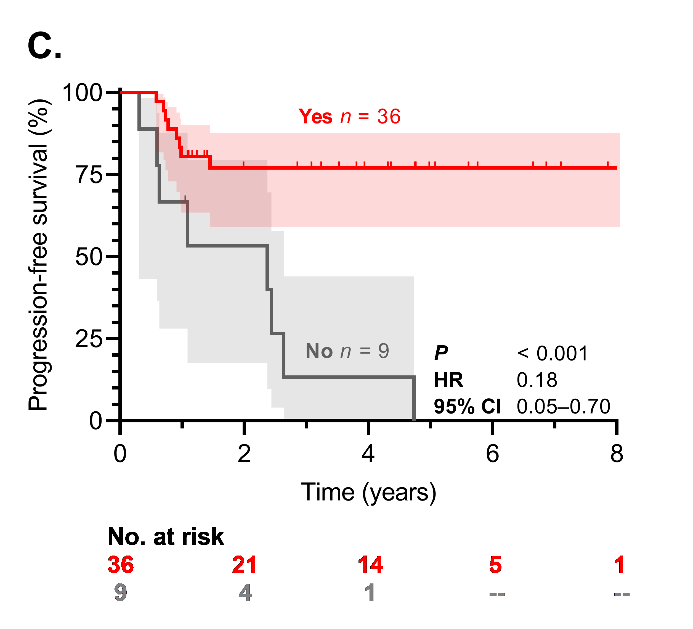

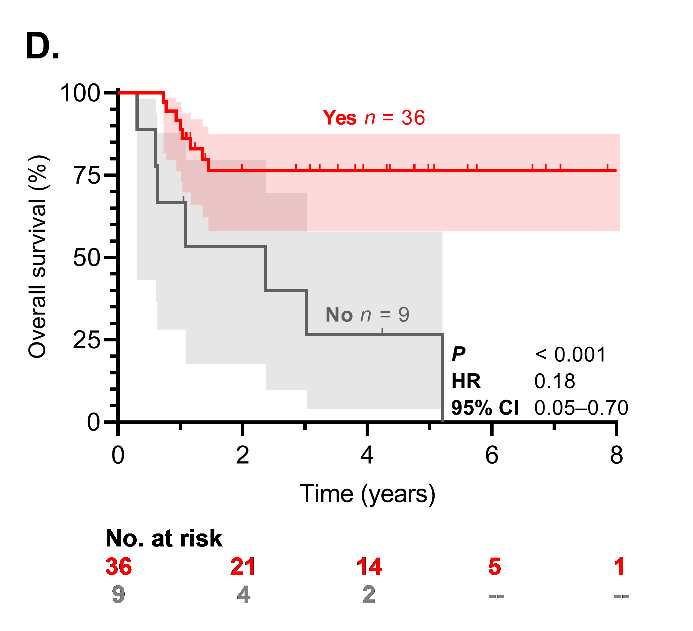


**Supplementary Figure 4.** Survival of the MATRix-IC (*n* = 78) versus MATRix-non-IC (*n* = 10) patients (**A.** progression-free survival, **B**. overall survival).

*CI, confidence interval; HR, hazard ratio; IC, inclusion criteria.*


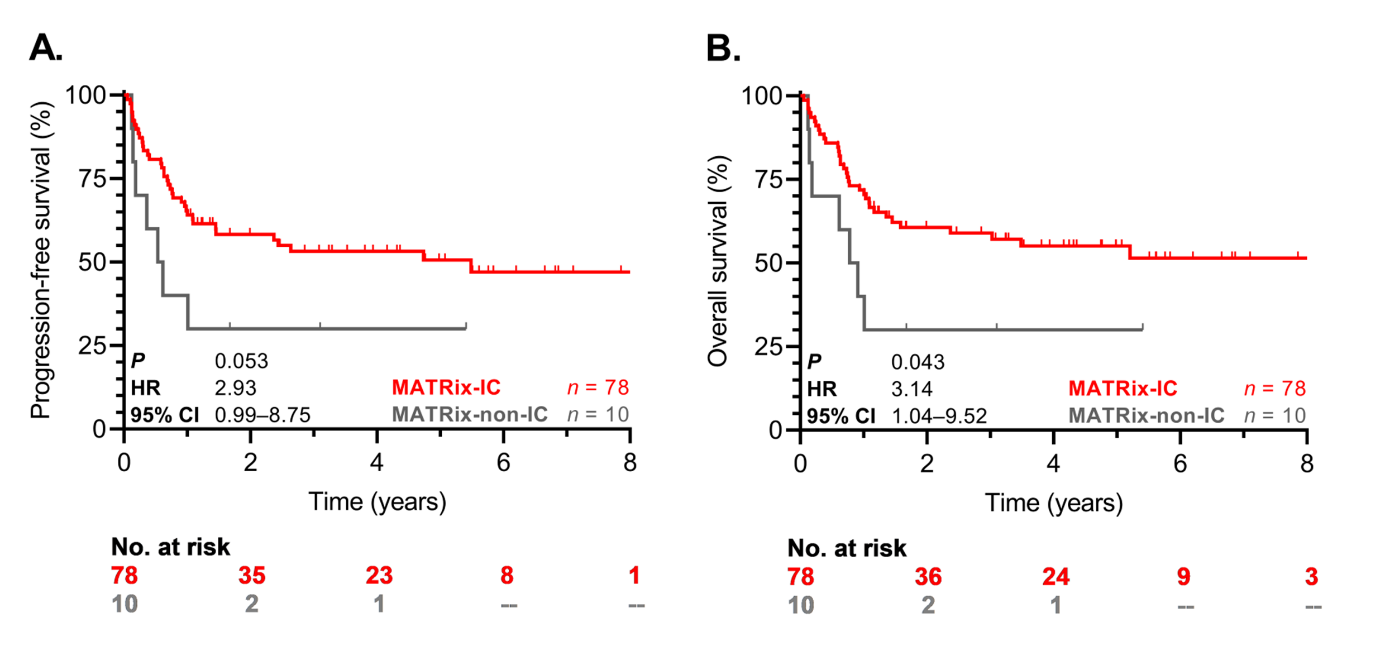

Supplement: Supplementary file 1 — Supporting Information S1 [file HON-43-e70142-s001.docx]
